# Comparison of changes in fecal microbiota of calves with and without dam

**DOI:** 10.7717/peerj.12826

**Published:** 2022-04-01

**Authors:** Mengya Li, Zhisheng Wang, Lizhi Wang, Bai Xue, Rui Hu, Huawei Zou, Siqiang Liu, Ali Mujtaba Shah, Quanhui Peng

**Affiliations:** Key Laboratory of Bovine Low-Carbon Farming and Safety Production, Sichuan Agricultural University, Sichuan Agricultural University, Chengdu, China

**Keywords:** Calf, Cultivation method, Microbial community, Dynamics

## Abstract

In pastoral areas and semi-agricultural and semi-pastoral areas of Sichuan, beef cattle breeding mode is mainly dependent on nature to raise livestock. On the one hand, owing to the shortage of forage grass in spring, cows suffer from malnutrition. On the other hand, competition for milk between human and livestock further deepens the malnutrition of newborn calves, and the mortality rate even exceeds 40%, resulting in serious waste of beef cattle source resources. The objective of this study was to investigate the effect of different cultivation methods (calves with and without dam) and age on calves hindgut microbiome. Sixteen healthy calves (Yak ♂ × Pian cattle ♀, with similar birthday 0 ± 2 d and body weight 13.1 ± 1.13 kg), were selected and randomly divided into two groups. The control group was cultivated with heifers, whereas the treatment group was cultivated without heifers and was fed milk replacer during the whole 95 days formal experimental period. Fecal samples were collected on 35, 65 and 95 days of age for high-throughput sequencing. The α-diversity was different between the two groups on day 35; however, the bacterial species richness and diversity was almost not different on day 95. Principal coordinates analysis revealed significant difference between the two groups on all the three time points, and the timepoints of day 65 and 95 were closer and separated from the timepoints of day 35 in calves with dam, whereas the timepoints of day 35 and 65 were closer and separated from day 95 in calves without dam. As time passed, the abundance of Firmicutes increased, while Proteobacteria and Actinobacteria decreased in calves with dam. But in calves without dam, the abundance of Bacteroidetes and Proteobacteria increased on day 65 and then decreased on day 95. In genus level, the relative abundance of *Bacteroides* decreased in calf with dam while its abundance increased first and then decreased in calf without dam but both resulted in the range of 3.5~4.5%. The relative abundance of *Lactobacillus* decreased, whereas *Ruminococcaceae UCG-005* increased in both groups as the calf grew up. It was concluded that the richness and evenness of the microbial communities was higher in calves with dam than without dam, and a stable gut microbiome in calve with dam is established earlier than calf without dam.

## Introduction

The beef industry of China has been challenged with the high rate of preweaned calf mortality (10~15%), and one of the major reasons in neonatal calves is enteric infections (Cho & Yoon, 2014; Uetake, 2013). Compared with healthy calves, calves with pneumonia and neonatal diarrhea have a lower bacterial diversity (Cho & Yoon, 2014; Uetake, 2013), indicting a possible link between gut microbiota and host health. Therefore, one of the most important way to reduce the prevalence of enteric infections is to improve gut microbiota development and composition. The use of rodent models has successfully revealed that the gut microbiota is associated with inflammatory bowel disease. More and more studies focus on the importance of gut microbiota in the health (for example, immune system) and production of livestock exist (Kogut & Arsenault, 2016; Malmuthuge, Griebel & Guan, 2015a; Du et al., 2015; Sommer & Bäckhed, 2013).

The gastrointestinal tracts (GIT) of newborns contain a less diverse of microbiome than those of adults, and progressive colonization over time increases microbial diversity (Thompson et al., 2015; Turroni et al., 2012). The microbial colonization of ruminant GIT begins at birth and gets microbes from animals’ surrounding, including diet of maternal colostrum and calves live together (Gerrits, 2019). Studies have shown that diet had a substantial influence on the composition of the calves’ gastrointestinal microbiota (Caporaso et al., 2010; Maslowski & Mackay, 2011), and colostrum was a rich microbial consortium which accelerates the bacterial colonization in the calf small intestine (Malmuthuge et al., 2015b). Studies have found that receiving breast milk is the most important factor related to the structure of the microbiome, which may affect subsequent immune and growth performance (Stewart et al., 2018). Fischer et al. (2018a, 2018b) have shown that fed heat-treated colostrum may serve as prebiotic to microbiota in the intestine of the neonatal calf, and delaying colostrum feeding within 12 h of life delay the colonization of bacteria in the intestine, possibly leaving the calf vulnerable to infections during the preweaning period. Many studies focused on effect of weaning methods (Dong et al., 2019; Meale et al., 2016) or feeding strategies (Kim et al., 2016) on gut microbiota of calves are available; So far, few studies have compared the changes in the gastrointestinal microbes of calves fed with breast milk and milk replacers at the same time, which can increase our understanding of calves before weaning.

There were some advantages on calves with dam; for example, that making use of milk can reduce feed cost. But the rearing method of calves with dam affected the reproduction rate of cows, and lack of concentrate intake limits the growth of calves. Most of the previous studies on intestinal microbiota in newborn pre-ruminants have mostly focused on fecal microbiota as they are thought to be representative of the hindgut microbiota (Klein-Jöbstl et al., 2014; Malmuthuge & Guan, 2017; McGarvey et al., 2010). The objective of this study was to compare the difference and change of fecal microbiota composition between calves with and without dam.

## Materials and Methods

The experimental protocol used in the present study was approved by the Animal Policy and Welfare Committee of the Agricultural Research Organization of Sichuan Province, China, and was in accordance with the guidelines of the Animal Care and Ethical Committee of the Sichuan Agricultural University (Permission code SYXK (chuan) 2014-187).

### Animals and experimental design

A total of 16 healthy calves with similar birthday (0 ± 2 d) and body weight (13.1 ± 1.13 kg) (Yak ♂ × Pian cattle ♀) were selected and randomly assigned to one of two groups according to their cultivation method. The control group was cultivated with heifers, whereas the other group was fed milk replacer. Milk replacer and warm boiled water (50–60 °C) in a ratio of 1:8. The milk replacer was washed and thoroughly stirred, then cold boiled water added and the water temperature adjusted to 37 °C. The daily feeding amount of milk replacer is 3% of body weight, and adjusted at any time with the increase of body weight. After 10 days of age, the control group followed the cows to pick small amounts of the TMR feed, and the treatment group was provided free access to the same diet. The diet was formulated according to China Feeding Standard of Beef Cattle (NY/T 815-2004) and the animal was fed twice daily at 07:00 and 17:00. After 3 days of adaptation period, the formal experiment lasted 90 days. On day 35, 65 and 95, the feces of calves were collected in 50 mL test tubes with lids. The samples were frozen immediately at −80 °C until use.

### DNA extraction

A total genomic DNA was extracted from fecal samples, according to the manufacturer’s instructions of DNeasy PowerSoil Kit (Qiagen, Valencia, CA, USA). After extraction, DNA integrity was detected by 0.8% agglutinate gel electrophoresis, and DNA concentration and purity were detected by NanoDrop ND-1000 spectrophotometer (Nyxor Pharmacia, Paris, France). All extracted DNA samples were stored at –20 °C until be used as templates for real-time PCR and Illumina sequence analyses.

### PCR amplification and 16s rRNA gene sequencing

A template, fecal total DNA, was amplified by the V4 variable of 16s rRNA genes. The universal primer set, 515F (5′-GTGCCAGCMGCCGCGGTAA-3′) and 806R (5′-GGACTACHVGGGTWTCTAAT-3′) are used (Caporaso et al., 2011). Specific primers with Barcode were used for PCR according to the selection of sequencing region. Each 25 μL system consisted of a 1 × PCR buffer, 1.5 mM MgCl2, each deoxynucleoside triphosphate at 0.4 μM, each primer at 1.0 μM, 0.5 U of KOD-Plus-Neo enzyme (Toyobo, Tokyo, Japan) and 10 ng of template DNA. And the PCR amplification procedure consisted of starting of denaturation at 94 °C for 1 min, then 30 cycles (including denaturation at 94 °C for 20 s, annealing at 54 °C for 30 s and elongation at 72 °C for 30 s) and a final extension 72 °C for 5 min. The PCR reactions were repeated three times for each sample and combined together. The PCR procedure was mixed with 1/6 volume of 6X loading buffer and detected by 2% agarose gel electrophoresis. We chose the sample between 200~400 bp, then the PCR production was purified using OMEGA Gel Extraction Kit (Omega Bio-Tek, Norcross, GA, USA), the gel purified barcoded amplicons were pooled with equal molar amount and quantified on a Qubit@ 2.0 Fluorometer (Thermo Scientific, Waltham, MA, USA). The library quality was assessed on the Qubit@ 2.0 Fluorometer (Thermo Scientific, Waltham, MA, USA) and Agilent Bioanalyzer 2100 system. Then, the pooled amplicons were paired-end sequenced (2 × 250 bp) on the Hiseq Illumina Sequencing Platform (Rhonin Biosciences Co., Ltd, Chengdu, China).

### Bioinformatics and statistical analysis

Paired-end reads from the original DNA fragments were merged using FLASH (Magoc & Salzberg, 2011). Paired-end reads was assigned to each sample according the unique barcode. The sequence with quality (length > 200 bp, without ambiguous base ‘N’, and average base quality score >30) were screened for chimmeras checking using Uchime (Edgar et al., 2011). Sequence were clustered into OTUs at 97% identity threshold using UPARSE algorithm (Edgar, 2013), and picked representative sequences for each OTU. Taxonomy was assigned using Silva database (Edgar, 2013) and the representative sequences were aligned using PyNAST (Caporaso et al., 2010). The analysis of alpha, which included calcuation of Chao1, ACE, Shannon and Simpson indices were conducted with Vegan (Version 2.0-2.R CRAN packet) and PD index was conducted with Picante (Kembel et al., 2010). Rarefaction curves were generated based on these metrics. Principal Co-ordinates analysis (PCoA) was performed to assess significant different between samples. Linear discriminant analysis (LDA) effect size (LEdSe) method was performed to identify the bacterial taxa differently represent between groups at genus or higher taxonomy level, which would help discover biomarkers (Segata et al., 2011). All statistical analysis was run using SAS v9.4 (SAS Institute Inc, Cary, NC, USA). The level of statistical significance was set at *P* < 0.05.

## Results

### Analysis of Illumina MiSeq sequencing data in feces of calves with and without dam

**Figure 1:**
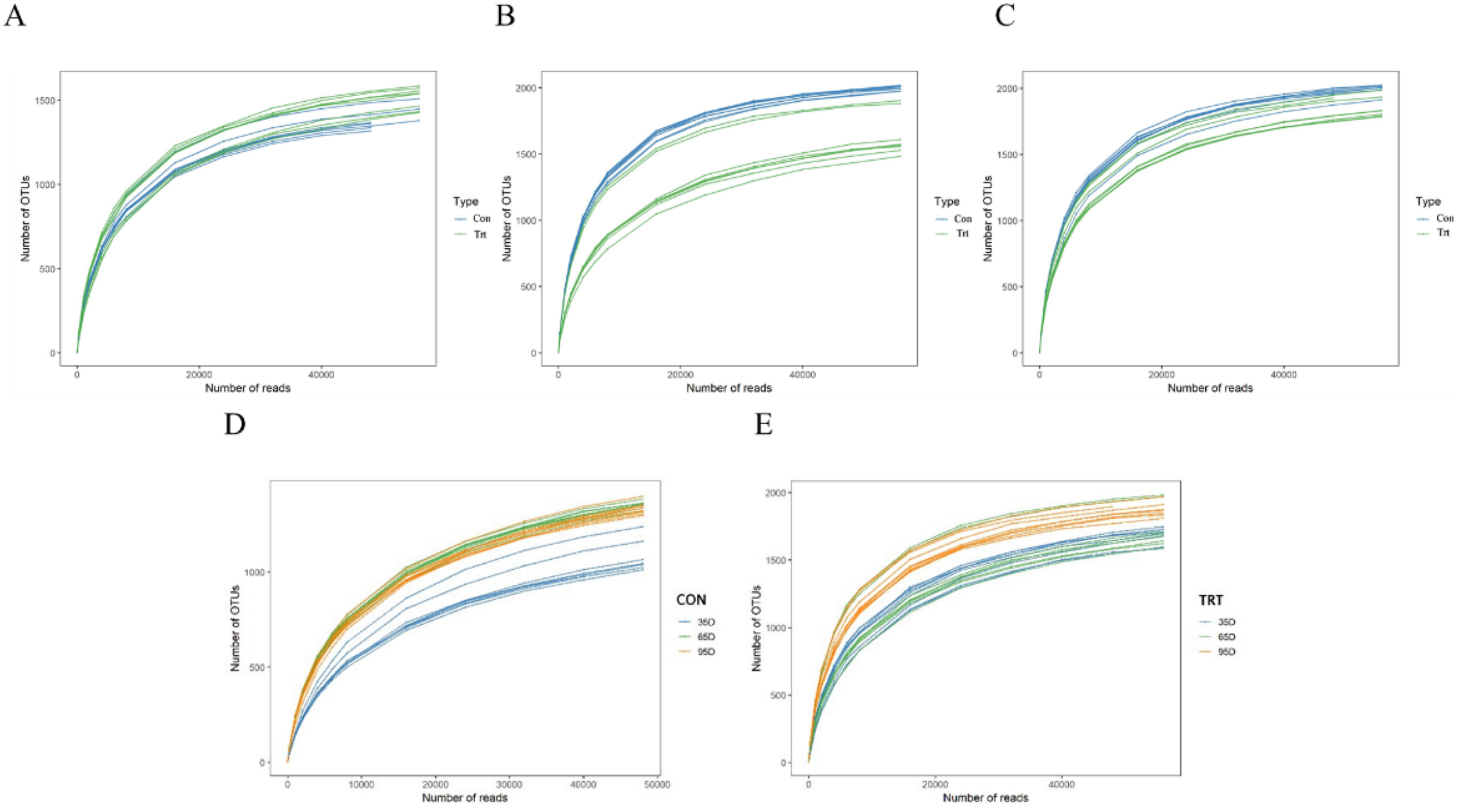
Rarefaction curves of bacterial communities between calves with and without dam (A–D). Con represented calves with dam and Trt represented calves without dam.

A rarefaction test was performed at the OTU level and the results are presented (Fig. 1). All the curves asymptotically approached a plateau, which suggested that new phylotypes could not be detected even if additional sequencing was performed, and the majority of the bacterial phylotypes in feces were identified.

Illumina MiSeq sequence of the 16S rRNA yielded 2,624,000 valid sequences from the 48 samples (including eight calves with dam and eight calves without dam, and samples were collected on day 35, 65 and 95 respectively), with a mean of 54,667 sequences per sample. These sequences were clustered into 62,010 operational taxonomic units (OTUs) at the 97% similarity level, with an average of 1,292 OTUs per sample.

**Figure 2:**
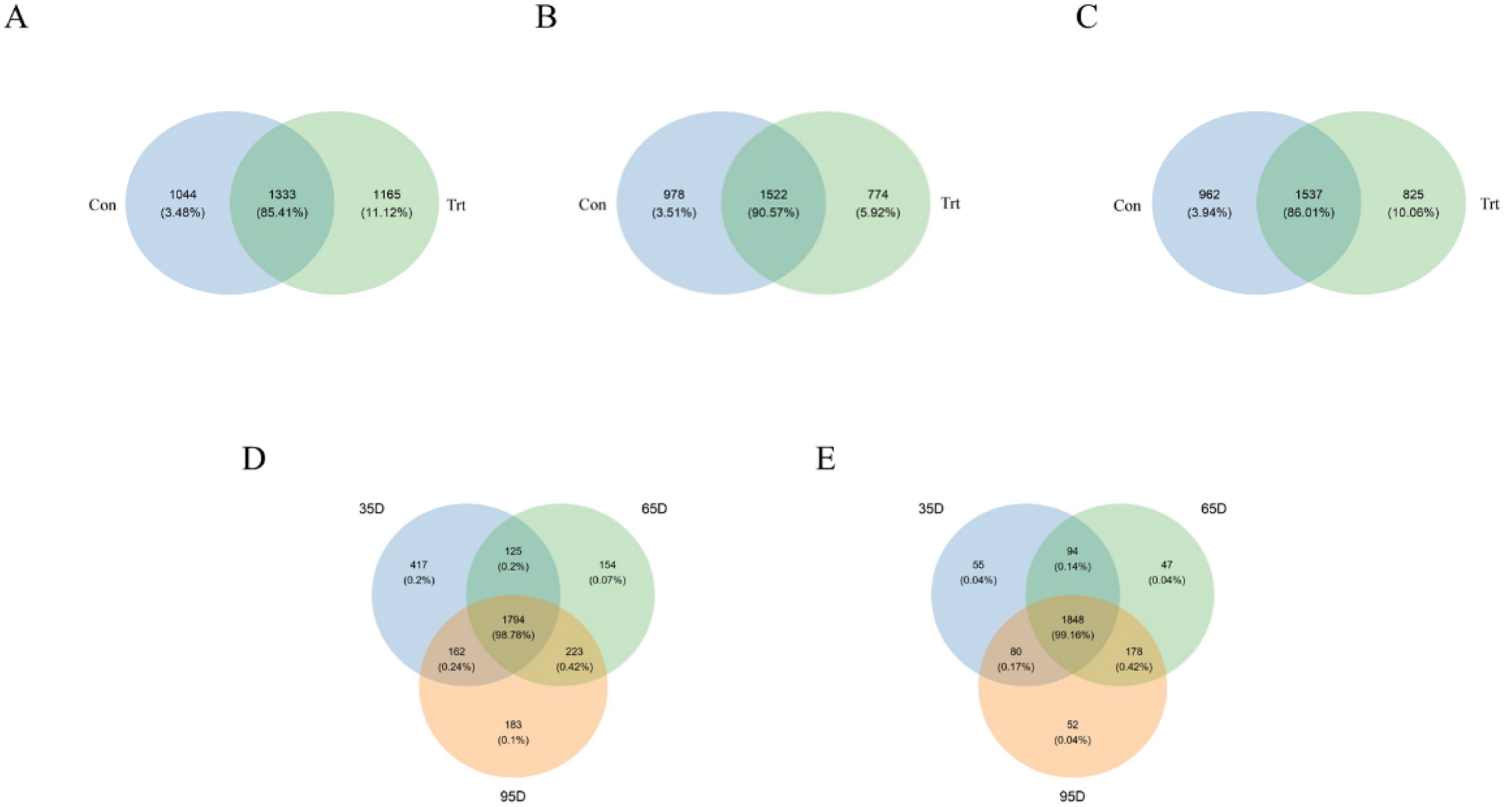
Venn diagram representation of the shared and exclusive bacterial OTUs at 97% similarity level between calves with and without dam (A–E). On day 35 (A), 65 (B) and 95 (C) and on the three timepoints of feces in calves with and without dam (D and E). Con represented calves with dam and Trt represented calves without dam.

According to the Venn diagram at a 97% similarity level (Fig. 2), the shared OTUs between the calves with and without dam was 1,333 (85.41% of sequence, Fig. 2A), the OTUs unique to the calves without dam was 1,165 (11.12% of sequence, Fig. 2A) on day 35. On day 65, the shared OTUs between calves with and without dam was 1,522 (90.57% of sequence, Fig. 2B), the OTUs unique to the calves without dam was 774 (5.92% of sequence, Fig. 2B). On day 95, the shared OTUs between calves with and without dam was 1,537 (86.01% of sequence, Fig. 2C), the OTUs unique to the calves without dam was 825 (10.06% of sequence, Fig. 2C). A total of 1,848 and 1,794 OTUs (99.16% and 98.78% of the total sequences) were common to calves with and without dam in three different timepoints, respectively. In calves with dam, the OTUs unique to day 35, 65 and 95 were 55 (0.04% of sequence), 47 (0.04% of sequence) and 52 (0.04% of sequence), respectively (Fig. 2D). In the meantime, the OTUs unique to day 35, 65 and 95 were 417 (0.2% of sequence), 154 (0.07% of sequence) and 183 (0.1% of sequence) in calves without dam (Fig. 2E).

The OTUs, species richness and diversity were estimated using the Chao1, ACE, Shannon and Simpson indices are shown in Table 1. On day 35, The PD indices of calves without dam was higher than calves with dam (*P* < 0.05). No significant difference was observed in the Chao1, ACE, Simpson and Shannon indices between the two groups (*P* > 0.05). On day 65, all the α-diversity indices including the Chao1, ACE, Simpson, Shannon and PD indices of calves without dam were lower than calves with dam (*P* < 0.01). However, on day 95, only the Chao1 and ACE indices of calves without dam were lower than calves with dam (*P* < 0.01).

**Table 1:** Summary of the Illumina MiSeq sequences data and statistical analysis of bacterial diversity in calves with and without dam on day 35, 65 and 95.

Item	Calves with dam			Calves without dam			*P*-value		
	35 D	65 D	95 D	35 D	65 D	95 D	Day (D)	Treatment (T)	D × T
OTUs	1,170.5	1,492.1^a^	1,453.8^a^	1,187.3	1,163.8^b^	1,283.9^b^	<0.001	0.011	<0.001
Chao1	1,408.0	1,782.1^a^	1,717.9^a^	1,495.3	1,424.9^b^	1,520.3^b^	0.001	0.036	<0.001
ACE	1,429.0	1,784.9^a^	1,720.7^a^	1,541.0	1,449.2^b^	1,542.8^b^	0.002	0.072	<0.001
Shannon	4.30	5.79^a^	5.60	4.40	4.89^b^	5.41	<0.001	<0.001	0.03
Simpson	0.93	0.99^a^	0.98	0.91	0.97^b^	0.99	0.015	0.27	0.636
PD	76.75^b^	88.94^a^	88.36	86.03^a^	81.39^b^	86.06	0.006	0.939	<0.001

**Note:**

Data are presented as mean of eight samples, means with different superscripts of minuscule represent significant different between the Calves with and without dam groups at the same time point.

In the time dimension, the α-diversity indices including the Chao1, ACE Simpson and PD indices of day 65 were greater than day 35 (*P* < 0.01), but no significant difference with day 95 in the calves with dam (*P* > 0.05). On the contrast, no significant difference on the Chao1, ACE and PD indices among the three timepoints was observed in the calves without dam (*P* > 0.05); however, the Shannon indices of day 65 and 95 were higher than that of day 35. These results indicated that the bacterial species richness and diversity in feces of calves with dam was greater than calves without dam in different timepoints, and the bacteria in the gastrointestinal of calves with dam is established earlier than calves without dam.

**Figure 3:**
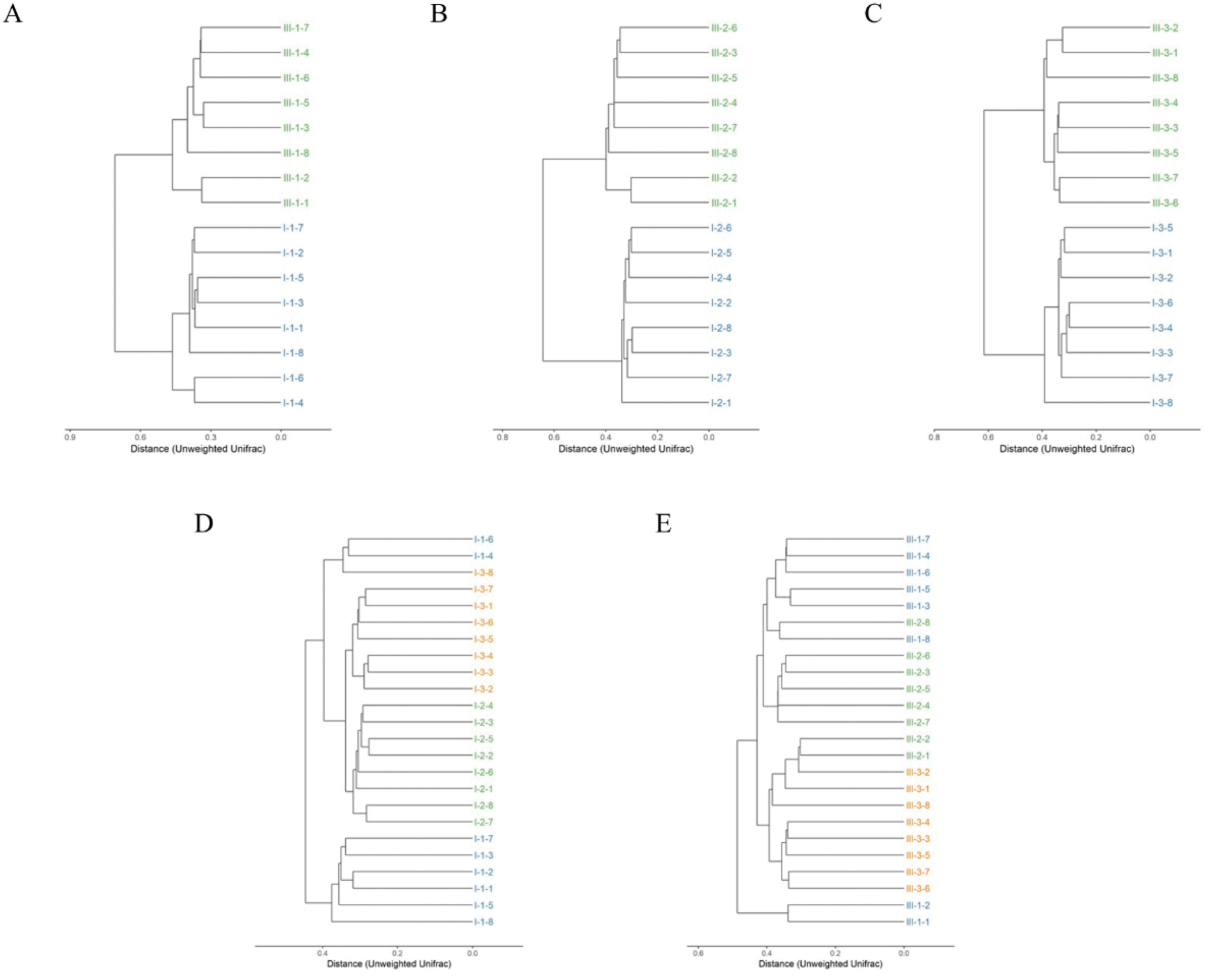
Hierarchical clustering of bacterial communities assessed using unweighted Unifrac metric analysis of OTUs between calves with and without dam. On day 35 (A), 65 (B) and 95 (C) and on the three timepoints of feces in calves with and without dam (D and E) at 97% similarity. The scale bar shows approximate unweighted UniFrac metric similarity coefficient of 0.2 in the comparison between calves with and without dam and 0.3 in the three different timepoints. Con represented calves with dam and Trt represented calves without dam. The Roman numeral I means the calves with dam, and III means the calves without dam.

Clustering of the samples based on unweighted analysis of OTUs at 97% identity is shown in Fig. 3. The closer the samples and the shorter the branches, indicating more similar the species composition of the two samples. Surprisingly, the samples were absolutely clustered into two different branches on day 35, 65 and 95 between calves with and without dam (Figs. 3A–3C), which means that the microbial composition was totally different in feces on the three different timepoints between the two groups.

The relationship of bacterial community among the three different timepoints showed that the samples were clustered into three branches (Fig. 3D). All eight samples of day 65 were clustered into the same clade, while two samples of day 35 (sample nos. 1-4 and 1-6) were clustered together with a sample of day 95 (sample nos. 3-8) into a seperate clade and then merged with samples of day 65 and 95 at the distance of less than 0.4 in calves with dam. This means that the microbial composition in calves with dam changed from day 35 on and kept stable thereafter. On the contrary, a totally different alteration trend was observed in the composition of calves without dam (Fig. 3E). All eight samples of day 95 and two samples of day 65 (sample nos. 2-1 and 2-2) were clustered into a clade at the distance of 0.4, while the samples of day 35 and 65 were clustered into one clade first and then mingled together at the distance over 0.4. Two samples of day 35 (sample nos. 1-1 and 1-2) were clustered into one clade and mingled with the left samples at the distance of over 0.5. This means that the microbial composition in calves without dam changed from day 65 on and kept stable thereafter. The evenness of microbial composition of calves with dam is superior than that of calves without dam.

**Figure 4:**
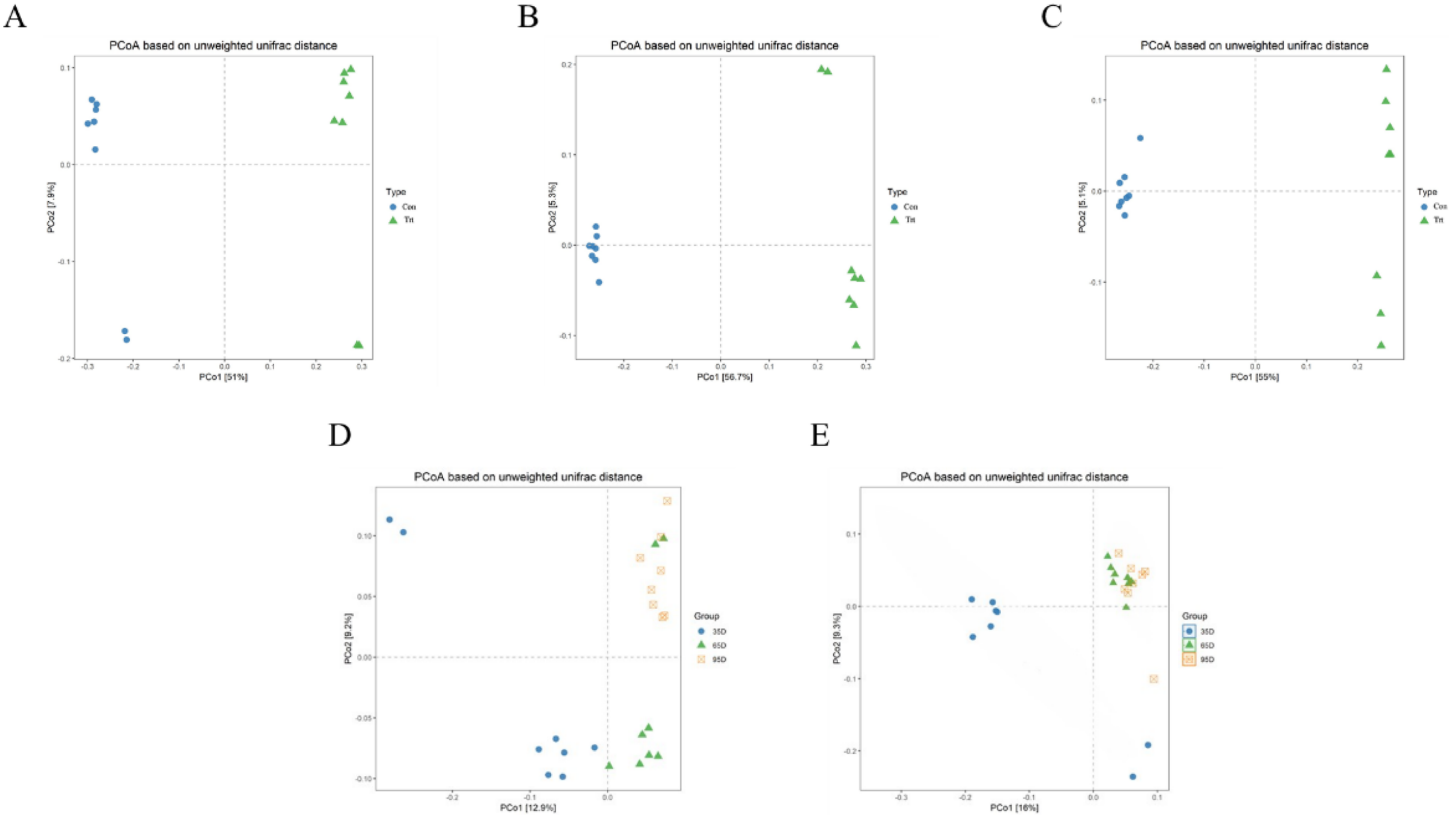
Principal Co-ordinates Analysis (PCoA) scores plot of fecal samples between calves with and without dam. On day 35 (A), 65 (B) and 95 (C) and on three timepoints in calves with and without dam (D and E) by an unweighted UniFrac analysis at the 97% similarity level. Con represented calves with dam and Trt represented calves without dam.

PCoA was conducted based on unweighted Unifrac distance to distinguish fecal microbial structure between the calves with and without dam on day 35, 65 and 95 respectively and the results are shown Fig. 4. The closer the distance between points the more similar the microbial community structure of the sample. The PCoA plot demonstrated that the microbial community between the calves with and without dam was different, and could be distinguished clearly (Figs. 4A–4C), and PCo1 accounted for 51%, 56.7% and 55% of variance on day 35, 65 and 95, respectively.

The characteristics of the PCoA of the three timepoints was that all 16 samples on day 65 and 95 were closely clustered together, while six samples on day 35 were vertically isolated from samples of day 65 and 95 in calves with dam (Fig. 4D), and the PC1 account for 16% of the variance. In terms of the samples of calves without dam, the samples of day 35 and 65 were horizontally distinguished from samples of day 95, but two exceptional plots of day 35 and 65 respectively existed (Fig. 4E), and the PC1 account for 12.9% of the variance. This again suggested that the establishment process of microbiota in calves with dam was earlier that calves without dam.

### Bacterial composition in feces between calves with and without dam

**Figure 5:**
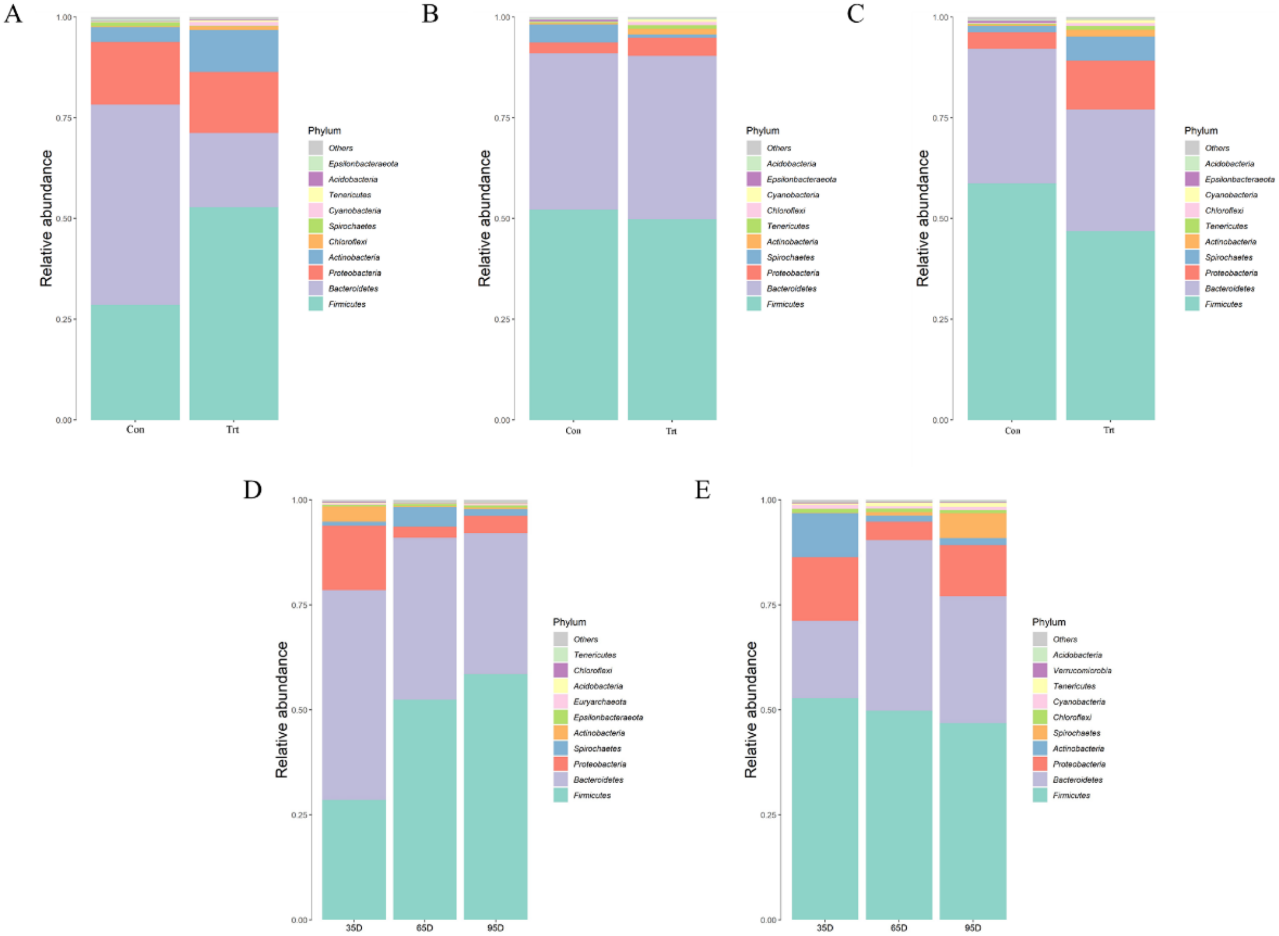
Relative abundance (%) of fecal bacteria at phyla level on three timepoints between calves with and without dam. On day 35 (A), 65 (B) and 95 (C) and on three timepoints in calves with and without dam (D and E). Con represented calves with dam and Trt represented calves without dam.

Species annotation and statistics were performed on different taxonomic levels using the effective sequences obtained Fig. 5. At the phylum level, the most common phyla in feces were Firmicutes, Bacteroidetes, Proteobacteria and Actinobacteria in calves with and without dam. Compared with calves with dam, only the abundance of Actinobacteria was higher than in calves without dam in the three different timepoints (*P* < 0.05). As time passed by, the abundance of Firmicutes in calves with dam increased, while Proteobacteria and Actinobacteria decreased (*P* < 0.05). The abundance of Bacteroidetes and Proteobacteria in calves without dam increased from day 35 to 65, and then decreased from day 65 to 95 (*P* < 0.05).

**Figure 6:**
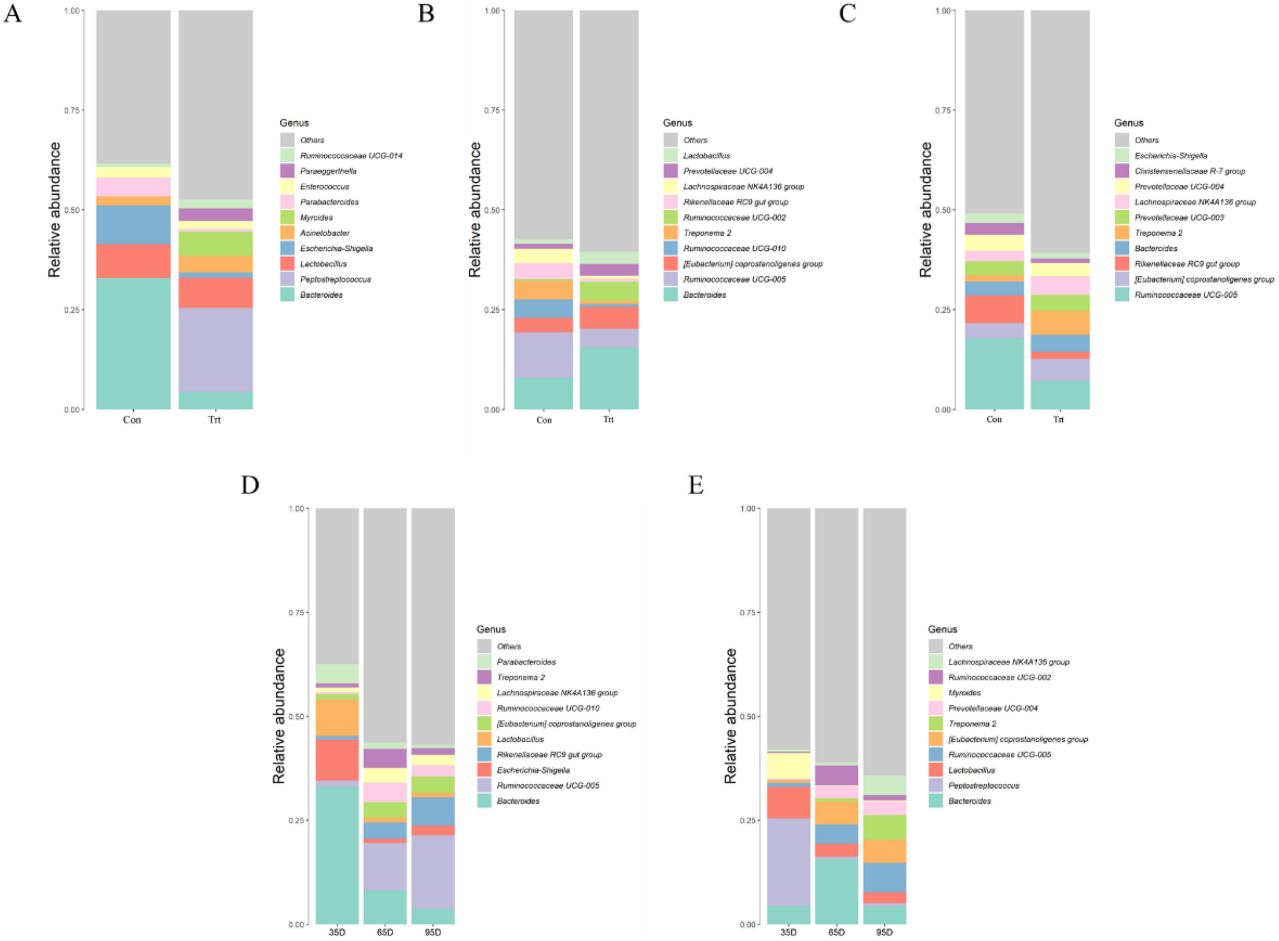
Relative abundance (%) of fecal bacteria at genus level on three timepoints between calves with and without dam. On day 35 (A), 65 (B) and 95 (C) and on three timepoints in calves with and without dam (D and E). Con represented calves with dam and Trt represented calves without dam.

At a lower taxonomical level, the top 10 were shown in Fig. 6. On day 35, the dominating genera (relative abundance ≥4%) in calves with dam were *Bacteroides* (32.91%), *Escherichia-Shigella* (9.79%), *Lactobacillus* (8.45%) and *Parabacteroides* (4.75%) while the genera *Peptostreptococcus* (21.05%), *Lactobacillus* (7.61%), *Myroides* (6.05%), *Bacteroides* (4.38%) and *Acinetobacter* (4.23%) were dominating in calves without dam. Compared with calves with dam, the abundance of *Bacteroides*, *Escherichia*-*Shigella* and *Parabacteroides* were lower and the abundance of *Peptostreptococcus*, *Myroides* and *Paraeggerthella* were higher in calves without dam (*P* < 0.05).

On day 65, the dominating genera (relative abundance ≥4%) in calves with dam were *Bacteroides* (7.96%), *Rikenellaceae RC9* (4.03%), *Treponema 2* (4.55%), *Ruminococcaceae UCG-010* (4.64%) and *Ruminococcaceae UCG-005* (11.33%), while the genera *Bacteroides* (15.59%), *Ruminococcaceae UCG-005* (4.62%), *[Eubacterium] coprostanoligenes group* (5.47%) and *Ruminococcaceae UCG-005* (5.47%) was dominating in calves without dam. Compared with calves with dam, the abundance of *Bacteroides*, *Lactobacillus*, and *Ruminococcaceae UCG-002* were higher (*P* < 0.05), while the genera *Ruminococcaceae UGG-005, Rikenellaceae RC9 gut group, Treponema 2 and Ruminococcaceae UCG-010* were lower in calves without dam (*P* < 0.05).

On day 95, the dominating genera (relative abundance ≥4%) in calves with dam were *Rikenellaceae RC9* (6.92%), *Prevotellaceae* (4.03%) and *Ruminococcaceae UCG-005* (17.8%), while the genera *Bacteroides* (4.33%), *Ruminococcaceae UCG-005* (7.12%), *[Eubacterium] coprostanoligenes group* (5.54%), *Lachnospiraceae NK4A136 group* (5.54%) and *Treponema 2* (5.97%) were dominating in calves without dam. Compared with calves with dam, the genera of *[Eubacterium] coprostanoligenes group* and *Treponema 2* were higher (*P* < 0.05) in calves without dam, while the genera *Ruminococcaceae UCG-005* and *Rikenellaceae RC9 gut group* were lower (*P* < 0.05).

## Discussion

The objective of the present study was to compare the fecal microbiota community composition between calves with and without dam from birth to 95 d of age, and the dynamics of fecal microbiota of the calves raised under different condition. To date, most of the previous studies focused on the effect of feeding colostrum (Fischer et al., 2018a) or milk replacer (Amado et al., 2019) on the calves. In present study, we compared the difference of microbial community in calves with and without dam.

A greater number of OTU was observed in calves with dam than calves without dam on day 65 and 95, indicating that a positive effect of heifers on the establishment of gastrointestinal microbiota. In addition, the Shannon and Simpson indices were greater in calves with dam than calves without dam on day 65, however no difference was obtained on day 95, suggesting that microflora of calves with dam was more complicated than calves without dam on day 65, although when the calves continue to grow up to 3 months of age, the GIT microflora become similar and kept stable.

Species richness and diversity is tightly relevant to different feeding strategies and ages. Several studies have shown that formula feeding considerably altered gut microbiota in early life (Dill-McFarland, Breaker & Suen, 2017; Kundu et al., 2017). Studies reported that claves fed milk replacer and starter had a higher Chao1 index in jejunum compared with calves with dam and became higher similar between 3 and 6 weeks, which caused by the introduction of a solid diet (Cui et al., 2020; Malmuthuge et al., 2019). On the other hand, some studies reported that the bacterial diversity of calf feces increased from 1 to 7 weeks whatever the feeding methods (Edrington et al., 2012; Oikonomou et al., 2013). The previous studies have reached a consensus that age is the main driving force in the establishment microbial communities of calf (Dill-McFarland, Breaker & Suen, 2017; Dill-McFarland et al., 2019; Jami et al., 2013). This was also mirrored in present study, the diversity and richness of fecal bacterial communities increased both in calves with and without dam suggesting a gradual establishment of a complex microbiome in GIT with age increased.

Calves fed pasteurized milk were reported in the previous study that have a similar fecal bacterial during the first 8 weeks of life, with significant differences between 1 and 2 years of age (Dill-McFarland, Breaker & Suen, 2017). Klein-Jöbstl et al. (2014) reported that the calf fecal communities became more similar after 11 weeks, indicated that the fecal microbial of calves will reach maturity at 3 months of age. In this study, the timepoints of day 65 and 95 were more similar to each other in calves with dam, whereas the timepoints of day 35 and 65 were more similar to each other in calves without dam in the PCoA polt. The current findings suggested that the establishment of microflora had a stable sequence, and the calves with dam establish microflora in GIT earlier than calves without dam.

Previous studies showed that three phyla (Firmicutes, Bacteroidetes and Proteobacteria) accounted for most reads from bovine rumen and feces (Jami et al., 2013; Klein-Jöbstl et al., 2014; Meale et al., 2016; Shanks et al., 2011). In our study the abundance of Firmicutes showed the highest overall and dominating in calves with and without dam on day 65 and 95, accounting for over 50% of the relative abundance, suggesting that Firmicutes play a critical role in the microbial ecology of the bovine gut. Foditsch et al. (2015) and Oikonomou et al. (2013) reported a high abundance of Firmicutes in rectal samples of dairy calves before weaning. It was also confirmed by Song et al. (2018) that the three predominant phyla in the hindgut mucosa-attached and digesta-attached microbiota of pre-weaned were Bacteroidete, Firmicutes and Proteobacteria in dairy calves within the first 42 d of life. Several studies have shown that the majority of the taxa belong to the Firmicutes in feces were about 55.2% (Shanks et al., 2011), 55% (Durso et al., 2011), 63.7% (Mao et al., 2012) and 63.41% (Kim et al., 2014). Bacteriodetes ranked the second with relative abundance 49.72% in calves with dam on day 35, whereas the Firmicutes was dominating in calves without dam. When the calves with dam grow up, Bacteriodetes decreased, while the relative abundance in calves without dam increased. No matter how it changes, its relative abundance (over 30%) remained the second in both groups. Our results are consistent with previous studies that a high abundance of Bacteroidetes plays an important role in maintaining normal intestinal physiology and function in calves (Foditsch et al., 2015; Klein-Jöbstl et al., 2014; Thompson et al., 2015).

*Bacteroides*, belong to obligate anaerobe, participated in and regulated the normal digestion and absorption of nutrients in animal gastrointestinal tract, which is beneficial to animal body. In present study, the genus abundance of *Bacteroides* in calves with dam was high on day 35 but decreased with age and to a similar level with calves without dam. At meantime, the abundance of *Bacteroides* in calves without dam increased on day 65 but decreased on day 95. The decrease of *Bacteroides* in calves with dam on day 65 and 95 compared with day 35 was similarly to the results reported by Song et al. (2018) observed in pre-weaned dairy calves. On day 95, the abundance of *Bacteroides* in both groups were in the range of 3~5%, this range may be the normal range for *Bacteroides* in mature cattle.

*Lactobacillus* is found as normal microbiota of the gastrointestinal tract and vaginal flora and have historically been thought to be a beneficial bacterium. *Lactobacillus* is important for the maintenance of healthy homeostasis as a probiotic (Shang et al., 2016), and it can improve the structure of intestinal flora and enhance intestinal barrier function, thereby enhancing the host’s immunity (Wang et al., 2015). In our study, *Lactobacillus* was found in fecal samples of calf with and without dam on day 35 at a relatively abundance over 7.5% and then decreased thereafter, which has a Similar trend with previously observed (Klein-Jöbstl et al., 2014; Malmuthuge et al., 2019; Uyeno, Sekiguchi & Kamagata, 2010). There were also studies showed that when calves grew up, the relative abundance of *Bacteroides* and *Lactobacillus* decreased while the abundance of *Paraprevotalla* increased (Dias et al., 2018; Dill-McFarland, Breaker & Suen, 2017). Malmuthuge et al. (2019) reported that the *Lacteobacillus*-dominated calves exhibited a higher expression of pro-inflammatory chemokines than the *Bacteroides*-dominanted calves. The decreased *Lactobacillus* may be one of the factor related to the decreased immune response when the calves grew up gradually.

Anaerobic bacteria can cause disease when they gain access to normally sterile sites, example *Peptostreptococcus* (Sykes, 2014). The genera *Peptostreptococcus* have shown to promotes colorectal carcinogenesis and may play a carcinogenic role through direct interaction with colonic epithelial cells (Long et al., 2019). And *Peptostreptococcus* may provokes a pro-inflammatory immune microenviron-ment permissive for colorectal tumorigenesis (Long et al., 2019). Klein-Jöbstl et al. (2014) reported that *Peptostreptococcus* was rarely detected in calves which were fed with colostrum and pasteurized whole milk. In this study, a high abundance of *Peptostreptococcus* was observed in calves without dam on day 35 and then almost disappeared on day 65 and 95. These results suggesting that the *Peptostreptococcus* could be an indicator of susceptible calves which warrants further investigation.

Studies have shown that family *Ruminococcaceae* was responsible for the degradation of diverse polysaccharides (Shang et al., 2016). Moreover, a higher recovery of *Ruminococcaceae* sequences was found in forage fed animals fecal samples than in grain fed animals (Callaway et al., 2010; Shanks et al., 2011). This fact implied that forage select microbial populations rich in cellulose decomposers. The increased abundance of *Ruminococcaceae UCG-005* may related with the amount of feed consumed by calves. In our study, the genus of Ruminococcaceae UCG-005 increased both in calves with and without dam when they grew up. Chen et al. (2020) reported that Ruminococcaceae UCG-005 had the highest abundance in feces of calves fed pasteurized milk and acidified milk on 90 days, which would improve the fiber utilization ability of calves. In addition, the increased abundance of *Ruminococcaceae UGG-005* (phylum Firmicutes) may again confirmed the important role of Firmicutes in the gastrointestinal tract.

In this study, fecal samples were collected at 35, 65 and 95 days of age of calves, and the time span was large. Rumen fluid could be collected weekly in subsequent studies to determine the microflora.

## Conclusion

In summary, the richness and evenness of fecal microbial community of calves without dam were lower than calves with dam, but the fecal microbial composition of calves with and without dam tended to be stable after 95 days of age, which is the theoretical foundation for calves weaning at 3 months of age. However, this study has shortcomings. For example, the time points for collecting fecal samples are calves 30, 60, and 90 days old, and the time span is relatively large. In the follow-up of this study, rumen fluid can be collected on a weekly basis and the microbial flora can be determined. In short, the results of this study can provide theoretical implications for raising calves in areas with harsh weather.

## Supplemental Information

10.7717/peerj.12826/supp-1 Supplemental Information 1 Ingredients and nutritional level of diet (DM basis).

## Additional Information and Declarations

### Data Availability

The following information was supplied regarding data availability: The data is available at NCBI: PRJNA706844.
