# Supplementary material for: Comparison of changes in fecal microbiota of calves with and without dam
Source: PeerJ. 2022 Apr 1;10:e12826. doi: 10.7717/peerj.12826 (PMC8978885; doi:10.7717/peerj.12826)
Supplement: Supplemental Information 1 [file peerj-10-12826-s001.docx]

Ingredients and nutritional level of diet (DM basis)

| **Ingredients** | **Percentage (％)** | **Item** | **Nutritional level** |
| --- | --- | --- | --- |
| Corn meal | 26.5 | NEmf/(MJ/kg) | 5.11 |
| Wheat bran | 3.5 | CP (%) | 12.00 |
| Soybean meal | 2.0 | ADF | 27.55 |
| Cottonseed meal | 5.3 | NDF | 47.77 |
| Urea | 0.2 | Ca (%) | 0.73 |
| Soy oil | 0.2 | Total P (%) | 0.48 |
| CaCO_3_ | 0.7 |  |  |
| CaHCO_3_ | 0.2 |  |  |
| Na_2_SO4 | 0.2 |  |  |
| NaCl | 0.5 |  |  |
| NaHCO_3_ | 0.4 |  |  |
| Premix^1)^ | 0.3 |  |  |
| Distilled grain | 30.00 |  |  |
| Hybrid giant napier | 30.00 |  |  |
| Total | 100 |  |  |

^1)^ The premix contains 8000 IU OF VA, 1000 IU of VD3, 50 mg of iron, 10 mg of copper, 60 mg of zinc, 40 mg of manganese, 0.2 mg of selenium, 0.2 mg of iodine and 0.1 mg of cobalt per kilogram.
